# Triglyceride-glucose index is a risk factor for breast cancer in China: a cross-sectional study

**DOI:** 10.1186/s12944-024-02008-0

**Published:** 2024-01-26

**Authors:** Jinghua Zhang, Binbin Yin, Ya Xi, Yongying Bai

**Affiliations:** 1 Department of Clinical Laboratory, Women’s Hospital, Zhejiang University School of Medicine, No. 1, Xueshi Road, Hangzhou, 31006 Zhejiang Province China; 2 Department of Central Laboratory, The Children’s Hospital, National Clinical Research Center of Medicine, Zhejiang University School of Medicine, National Clinical Research Center for Child Health, Hangzhou, 310051 China

**Keywords:** Triglyceride-glucose index, Insulin resistance, Breast cancer, Benign breast disease, Risk, Biomarker

## Abstract

**Background:**

This research delved into the association between the risk of the Chinese population suffering from breast cancer (BC) and the triglyceride-glucose (TyG) index.

**Methods:**

A total of 2,111 sufferers with benign breast disease (BBD) and 477 sufferers with BC were enrolled, and their TyG index was measured. Participants with varying TyG index values were categorized into quartiles. Logistic regression analysis was employed to assess the relationship between the TyG index and BC risk. The diagnostic performance of the TyG index for different stages of BC was measured using the receiver operating characteristic (ROC) curve.

**Results:**

The TyG index of BC sufferers exceeded that of BBD (*P* < 0.001). A continuous increase in the risk of BC was found to be positively correlated with an ever-increasing TyG index. In the unadjusted model, the risk of getting BC mounted with quartiles of the TyG index growing (*P* < 0.001). In a logistic regression analysis that included all confounders, the highest quartile of the TyG index was strongly linked to BC risk [1.43 (1.01, 2.02), *P* < 0.05]. Moreover, with the adjustment of potential confounders, a high TyG index was found to result in a 2.53-fold higher risk of being diagnosed with advanced BC.

**Conclusions:**

The risen TyG index was positively correlated to the heightening risk of BC and had the potential to serve as a promising biomarker for BC. Individuals with a high TyG index ought to be mindful of the heightened risk of BC onset and progression.

**Supplementary Information:**

The online version contains supplementary material available at 10.1186/s12944-024-02008-0.

## Background

Breast cancer (BC) poses a significant public health challenge as it is the most common cancer globally [1]. In 2020 alone, it was estimated that the quantity of breast cancer cases would be around 2.3 million, with 685,000 deaths, and the previous projections suggested these numbers would continue to rise [2, 3]. Identifying modifiable risk factors and high-risk individuals is crucial to reducing the social impact of breast cancer. Despite significant advancements in breast cancer screening, diagnosis, therapy, and recurrence monitoring in the past few decades [4], the incidence rate of BC continues to increase [5]. Therefore, developing practical and reliable non-invasive markers that can identify the risk of symptomatic individuals is of significance to facilitate early diagnosis and reduce morbidity and mortality.

Metabolic syndrome (MetS), characterized by a range of biological factors such as obesity, dyslipidemia, dysglycemia, and hypertension, shows a close link to various types of diseases, including cancer [6, 7]. In the case of breast cancer, MetS has been linked to high-risk diseases and a poorer prognosis [8, 9]. Insulin resistance (IR), an important component of MetS, is indispensable in the evolving process of breast cancer [10]. However, the hyperinsulinemic-euglycemic clamp, which is the gold standard way to measure IR, is invasive, inefficient, labor-intensive, and technically challenging [11, 12], limiting its applicability in clinical settings.

Recently, the triglyceride-glucose (TyG) index has emerged as a promising object in medical research. This index, calculated from triglyceride level and glucose level, acts as a novel proxy for labeling IR [13]. It is practical, effective, and reproducible, demonstrating good sensitivity and specificity in detecting IR [14–16]. Moreover, several researches have shown that the TyG index pertains to various cancers, and a positive correlation represented by the TyG index and BC risk has been notified in past studies conducted in Western populations and Southeast Asia [17–19]. It is worth noting that China accounts for 24% of newly diagnosed cases and 30% of BC deaths world-wide [20], and the median age of onset and diagnosis of BC is earlier than in Western countries and other Asian countries like Korea and Japan [21]. In conclusion, it is significant to consider unique genetic, environmental, and lifestyle factors in the Chinese population that may influence this relationship differently. Therefore, the research aimed to assess the plausible correlation between the TyG index and breast cancer related to the Chinese population, filling a significant research gap.

## Methods

### Study design and subjects

A retro analysis was made with 3,097 sufferers undergoing breast disease admitted to the Department of Surgery, Women’s Hospital, Zhejiang University School of Medicine, from March 2020 to November 2021. The study received approval from the hospital’s ethics committee (approval number: IRB-20,210,315-R), and informed consent was waived as anonymized sufferer records were used. However, certain individuals who meet the following criteria are excluded: (1) missing data on the TyG index, which consisted of triglyceride and blood glucose (BG) levels; (2) missing relevant demographic information or incomplete clinicopathological data; (3) duplicated data; (4) under 18 years of age; (5) male participants; (6) individuals who received surgical treatments, chemotherapy, or radiotherapy before enrollment; (7) individuals with a history of cancer or autoimmune disorders; (8) individuals with conditions that may affect blood glucose levels, such as Cushing’s syndrome, hyperthyroidism, polycystic ovary syndrome, diabetes mellitus, or pancreatitis; (9) individuals using fenofibrate triglyceride-lowering drugs; (10) pregnant or breastfeeding women.

**Fig. 1:**
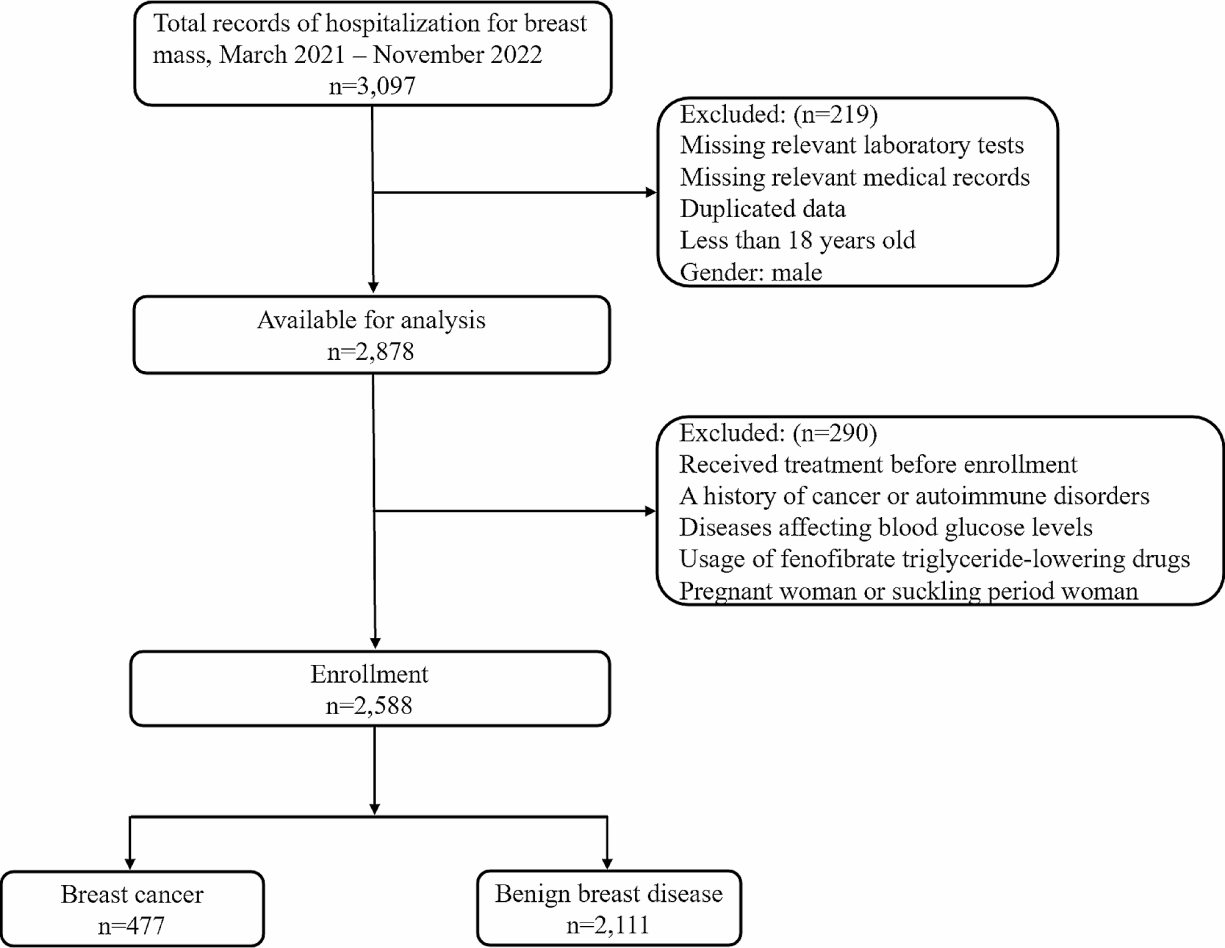
Flow chart of study subjects

The final analysis included a total of 2,588 individuals, comprising 2,111 cases of benign breast diseases (81.57%) and 477 cases of breast cancer (18.43%). The diagnosis of benign breast diseases or breast cancer in all enrolled sufferers was confirmed by postoperative pathology, and the staging guideline developed by the American Joint Committee on Cancer was exploited to determine the BC stages [22]. The screening details for the participants were illustrated in Fig. 1.

### Data collection

The demographics of the participants were collected from the hospital information system, which included data on sex, age, weight, height, smoking, alcohol consumption, familial record of malignancy, age at menarche, hormonal contraception, and comorbidities. The laboratory information system provided measurements of total cholesterol (TC), triglyceride (TG), high-density lipoprotein cholesterol (HDL-c), low-density lipoprotein cholesterol (LDL-c), and blood glucose.

### Laboratory analysis

The concentrations of TC, TG, HDL-c, LDL-c, and BG were analyzed utilizing an AU5800 chemistry analyzer (Beckman-Coulter, USA) in the hospital’s clinical laboratory. The instrument underwent daily internal quality controls and annual calibration to ensure accurate and reliable results. All procedures were executed in accordance with the instrument’s standard operating procedures to maintain consistency and accuracy.

### Definition of index

The formula for computing indexes was exemplified:

Body mass index (BMI) = weight (kg)/height squared (m^2^). BMI was divided into underweight, normal weight, overweight, or obese, corresponding to < 18.5, 18.5–23.9, 24–28, and > 28 kg/m^2^, respectively [23].

TyG index = Ln [TG (mg/dl) × BG (mg/dl)/2] [16].

### Statistical analysis

Statistical analyses were acquired through IBM SPSS 20.0 (Chicago, USA), and figures were obtained through GraphPad Prism 8.0 (California, USA). Continuous variables in the study were illustrated using means ± standard deviations (means ± SD), while categorical variables were reported using frequencies and proportions (n, %). The independent-sample *t*-test, or one-way analysis of variance, was employed. The chi-square test was utilized for comparing categorical variables across groups. The diagnostic characteristics of the TyG index for different stages of BC were measured considering the receiver operating characteristic (ROC) curve and the accompanying area under the curve (AUC). Additionally, the nonlinearity of the dose-response curve was evaluated to explore the correlation between the TyG index and BC risk. Five knots were created at the 10th, 25th, 50th, 75th, and 90th percentiles, with the 50th percentile’s TyG index as a reference point. Odds ratios (ORs) and 95% confidence intervals (CIs) for each TyG index quartile were determined using analyses of logistic regression, with or without adjustments for potential covariates. *P* < 0.05 was denoted as statistical significance.

## Results

### Basic features of the enrolled sufferers

Table 1 presented the basic features of the enrolled sufferers. Among the 2,588 sufferers, the mean age was 42.17 ± 11.78 years, and the average BMI was 22.3 ± 3.0 kg/m^2^. Out of the individuals affected, 477 sufferers were diagnosed with breast cancer, including 101 (21.17%) with carcinoma in situ (CIS) and 376 (78.83%) with invasive breast cancer. Significant intergroup discrepancies existed in the BC and BBD groups in terms of age, height, weight, BMI, TC, TG, LDL-c, BG, TyG index, and age at menarche (all *P* < 0.001). Additionally, the percentages of BMI category, TyG index category, smoking status, alcohol status, hypertension, and family history of cancer exhibited substantial disparities between the two groups (all *P* < 0.05). However, there was no intergroup difference in HDL-c levels or hormonal contraception rate (all *P* > 0.05).

**Table 1:** Clinical characteristics of participants

Variables	Overall	BC (+)	BC (-)	*P*-value
**n**	2,588	477	2,111	
**Age** (years)	42.17 ± 11.78	51.40 ± 10.68	40.09 ± 11.00	< 0.001
**Height** (cm)	159.7 ± 5.0	158.6 ± 5.1	160.0 ± 4.9	< 0.001
**Weight** (kg)	57.0 ± 8.2	58.4 ± 8.5	56.7 ± 8.1	< 0.001
**BMI** (kg/m^2^)	22.3 ± 3.0	23.2 ± 3.1	22.1 ± 3.0	< 0.001
Underweight	209 (8.08)	16 (3.35)	193 (9.14)	
Normal	1722 (66.54)	293 (61.43)	1429 (67.70)	
Overweight	541 (20.90)	135 (28.30)	406 (19.23)	
Obese	116 (4.48)	33 (6.92)	83 (3.93)	
**TG** (mmol/L)	1.23 ± 0.77	1.36 ± 0.74	1.21 ± 0.77	< 0.001
**TC** (mmol/L)	4.66 ± 0.97	4.93 ± 1.04	4.60 ± 0.94	< 0.001
**HDL-c** (mmol/L)	1.33 ± 0.47	1.33 ± 0.48	1.33 ± 0.47	0.839
**LDL-c** (mmol/L)	2.80 ± 0.73	2.99 ± 0.77	2.75 ± 0.72	< 0.001
**BG** (mmol/L)	4.95 ± 0.80	5.14 ± 0.86	4.90 ± 0.78	< 0.001
**TyG index**	8.28 ± 0.57	8.44 ± 0.55	8.24 ± 0.57	< 0.001
Q1 (6.88–7.87)	655 (25.31)	67 (14.05)	588 (27.85)	
Q2 (7.88–8.20)	643 (24.85)	110 (23.06)	533 (25.25)	
Q3 (8.21–8.62)	651 (25.15)	130 (27.25)	521 (24.68)	
Q4 (8.63–10.89)	639 (24.69)	170 (35.64)	469 (22.22)	
**Smoking** (%)	46 (1.78)	16 (3.35)	30 (1.42)	0.004
**Alcohol** (%)	71 (2.74)	20 (4.19)	51 (2.42)	0.032
**Hypertension** (%)	261 (10.09)	91 (19.08)	170 (8.05)	< 0.001
**Family history of cancer** (%)	409 (15.80)	104 (21.80)	305 (14.45)	< 0.001
**Age at menarche** (years)	14.35 ± 1.67	14.78 ± 1.73	14.26 ± 1.64	< 0.001
**Hormonal contraception** (%)	20 (0.77)	3 (0.63)	17 (0.81)	0.691

Values are expressed as mean ± standard deviation or number (percent)

Abbreviations: BC (+), breast cancer; BC (-), benign breast disease; BMI, body mass index; TG, triglyceride; TC, total cholesterol; HDL-c, high-density lipoprotein cholesterol; LDL-c, low-density lipoprotein cholesterol; BG, blood glucose; TyG index, triglyceride-glucose index; Q: quartile

### The BC risk and the TyG index

**Fig. 2:**
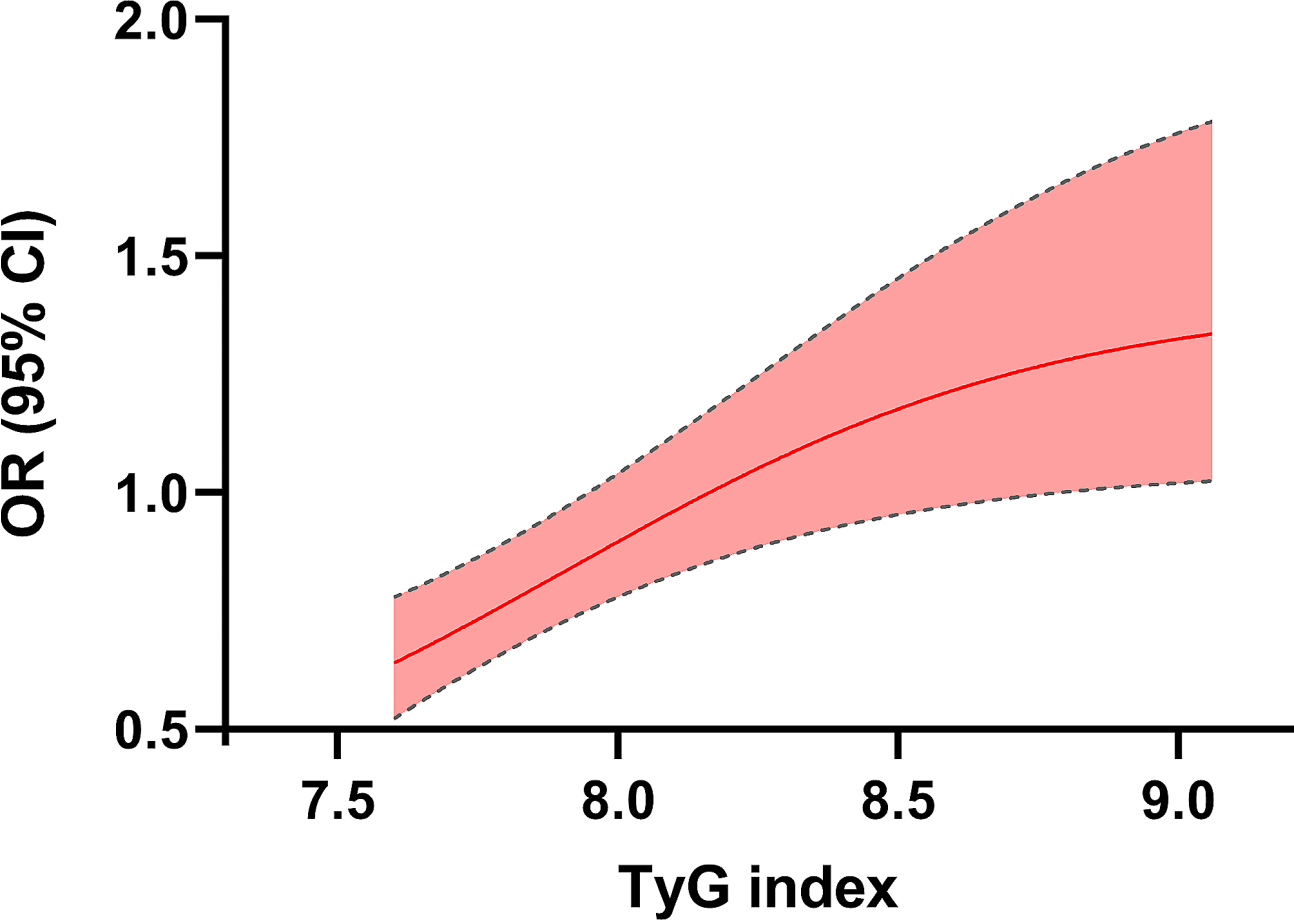
A nonlinear relationship of the TyG index with the risk of breast cancer using a dose-response curve with 5 knots. Cubic spline graph of the unadjusted ORs (represented by the solid line) and 95% CI (represented by the dotted line)

Figure 2; Table 2 illustrated the link between the BC risk and the TyG index. Using the 50th percentile’s TyG index as a reference, the results in Fig. 2 indicated a positive dose-response connection between BC risk and the TyG index in the total population. Crude and adjusted risks for the correlation of TyG index quartiles and BC risk were specified in Table 2. In the unadjusted Model 1, individuals’ TyG index in the quartiles of 2nd (Q2), 3rd (Q3), and 4th (Q4) showed higher risks of BC compared to the first quartile (Q1), with corresponding ORs (95% CI) of 1.81 (1.31, 2.51), 2.19 (1.59, 3.01), and 3.18 (2.34, 4.33), respectively.

**Table 2:** Odds ratios for the association of TyG index with BC risk

	Model 1		Model 2		Model 3	
	OR (95% CI)	*P*-value	OR (95% CI)	*P*-value	OR (95% CI)	*P*-value
**Age** (years)	-	-	4.50 (3.50, 5.78)	< 0.001	4.15 (3.18, 5.41)	< 0.001
**BMI** (kg/m^2^)	-	-	1.18 (1.00, 1.39)	0.056	1.15 (0.98, 1.36)	0.093
**Smoking** (%)	-	-	-	-	2.55 (1.11, 5.86)	0.027
**Alcohol** (%)	-	-	-	-	0.75 (0.37, 1.52)	0.421
**Hypertension** (%)	-	-	-	-	1.35 (1.00, 1.81)	0.047
**Family history of cancer** (%)	-	-	-	-	1.17 (0.90, 1.53)	0.236
**Age at menarche** (years)	-	-	-	-	1.07 (0.86, 1.33)	0.541
**Hormonal contraception**	-	-	-	-	0.95 (0.27, 3.43)	0.941
**TyG index**	-	-	-	-	-	-
Q1	Reference	-	Reference	-	Reference	-
Q2	1.81 (1.31, 2.51)	< 0.001	1.34 (0.95, 1.88)	0.095	1.32 (0.94, 1.85)	0.115
Q3	2.19 (1.59, 3.01)	< 0.001	1.32 (0.94, 1.86)	0.104	1.30 (0.93, 1.83)	0.130
Q4	3.18 (2.34, 4.33)	< 0.001	1.50 (1.07, 2.11)	0.020	1.43 (1.01, 2.02)	0.041

Model 1 was unadjusted. Model 2 was adjusted for age and BMI. Model 3 was adjusted for age, BMI, smoking status, drinking status, hypertension, family history of malignancy, age at menarche, and hormonal contraception

Abbreviations: TyG, triglyceride-glucose; BC, breast cancer; OR, odds ratio; CI, confidence interval; BMI, body mass index; Q, quartile

In model 2, the Q4 of the TyG index showed a higher adjusted risk [1.50 (1.07, 2.11), *P* < 0.05] compared to the first quartile (Q1) after modifying age and BMI. Similarly, individuals in Q4 of the TyG index group in model 3 had a statistically significant higher risk, with an OR of [1.43 (1.01, 2.02), *P* < 0.05]. Model 3 included additional confounders regarding age, BMI, drinking, smoking, hypertension, family history of malignancy, age at menarche, and hormonal contraception.

### The TyG index in the BC stages

**Fig. 3:**
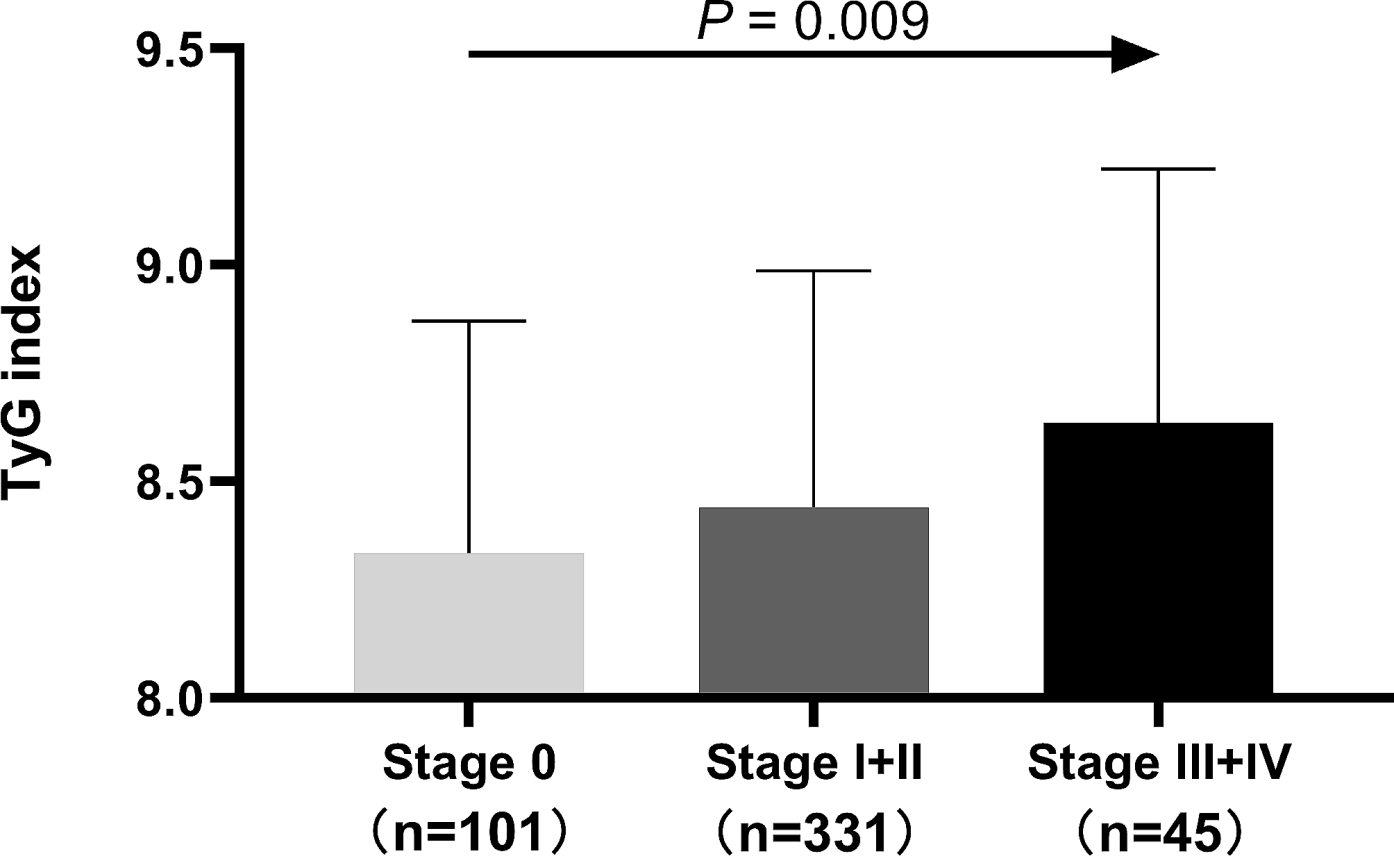
Comparison of the TyG index among different stages of BC. TyG: triglyceride-glucose; stage 0: carcinoma in situ

**Fig. 4:**
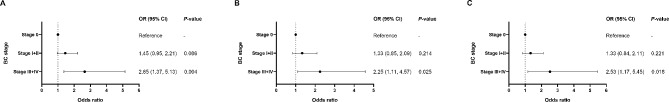
The relationship between the TyG index and breast cancer stages. (**A**) unadjusted; (**B**) adjusted for age and BMI; (**C**) adjusted for age, BMI, smoking status, drinking status, hypertension, family history of malignancy, age at menarche, and hormonal contraception. Logistic analysis was employed to assess the risk, with stage 0 serving as the reference point. BC: breast cancer; stage 0: carcinoma in situ; OR: odds ratio

Sufferers with advanced stages of the disease have a poorer prognosis. This study looked at the link between the TyG index and BC stages, which were categorized into stage 0 (carcinoma in situ), early stages (stages I + II), and advanced stages (stages III + IV). Figure 3 demonstrated a climbing trend in the expression of the TyG index from carcinoma in situ to advanced stages of BC (*P* < 0.01). Additionally, risk analysis was conducted on various stages of BC, using stage 0 as the reference. Figure 4 displayed the crude and adjusted ORs (95% CI) to investigate the correlation between the TyG index and the BC risk at various stages. In comparison with the TyG index for stage 0, the risk of advanced stages increased by 2.65 times in the unadjusted model (Fig. 4A). Similar results were obtained in models 2 (Fig. 4B) and 3 (Fig. 4C), which adjusted for age and BMI or included additional factors such as drinking status, smoking status, hypertension, family history of malignancy, age at menarche, and hormonal contraception.

### Diagnostic characteristic of the TyG index for BC

**Fig. 5:**
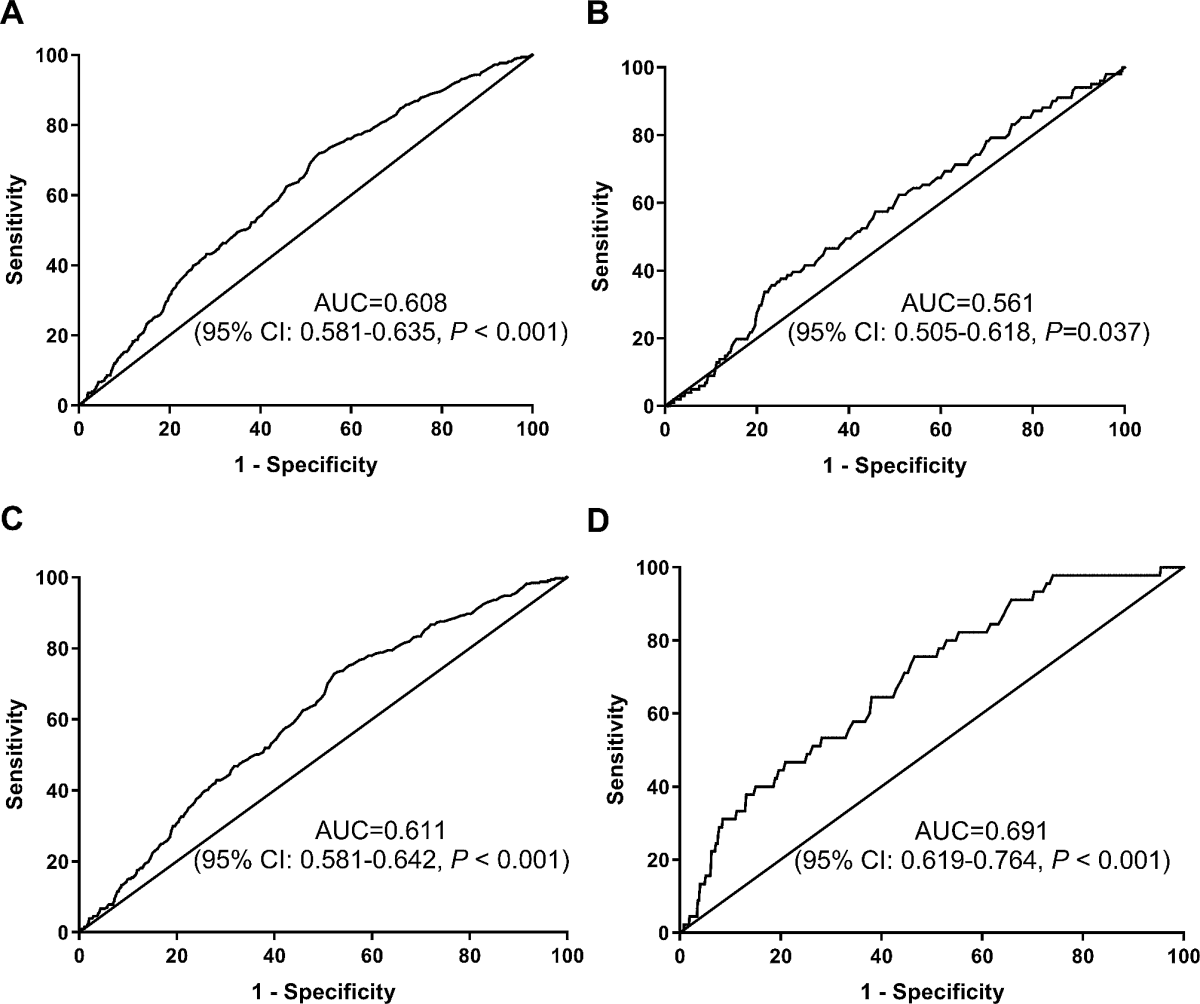
ROC of the TyG index in differentiating between benign and malignant breast diseases. (**A**) ROC of the TyG index in differentiating between benign andtotal population of breast cancer; (**B**) ROC of the TyG index in differentiating between benign and carcinoma in situ of breast cancer; (**C**) ROC of the TyG index in differentiating between benign and early stages of breast cancer; (**D**) ROC of the TyG index in differentiating between benign and advanced stages of breast cancer. ROC: receiver operative characteristic; TyG: triglyceride-glucose; AUC: area under the curve; CI: confidence interval

Figure 5 displayed the ROC curves for the TyG index in differentiating between benign and malignant breast diseases. The optimal threshold of the TyG index for BC was found to be 8.12 (AUC 0.608, sensitivity 71.91%, specificity 47.04%, *P* < 0.001) (Fig. 5A). The AUC for carcinoma in situ was 0.561 (sensitivity 35.64%, specificity 76.79%, *P* < 0.05) (Fig. 5B), while that for early stages was 0.611 (sensitivity 72.81%, specificity 47.75%, *P* < 0.001) (Fig. 5C). Interestingly, the TyG index of advanced stages had a greater maximum satisfactory accuracy and specificity than the other three groups, with an AUC of 0.691 (sensitivity 75.56%, specificity 53.43%, *P* < 0.001) (Fig. 5D).

## Discussion

In this study, the aim was to delve into the correlation between the TyG index and breast cancer. This study included 2,111 sufferers with benign breast disease and 477 sufferers with breast cancer, making it the first comprehensive study concentrating on the connection between the TyG index and breast cancer in Chinese residents. BC sufferers had notable levels of the TyG index than those with BBD. Furthermore, a positive interplay between the TyG index and BC risk was found in the unadjusted model. Even after adjustments for various confounding factors, a significantly increased BC risk was observed in individuals in the uppermost quartile of the TyG index. Interestingly, the TyG index levels were positively correlated with the risk of advanced stages of breast cancer, suggesting its potential as a non-invasive serum marker reflecting breast cancer development and progression.

Epidemiological evidence increasingly indicates a correlation between MetS and the evolving process of breast cancer. Additionally, this association is linked to an unfavorable treatment response, accelerated disease progression, and an unfavorable prognosis [8]. Insulin resistance, characterized by suppression of hepatic glucose manufacture and downward sensitivity to insulin-mediated glucose disposal [24], contributes greatly to the pathogenesis of MetS [10]. Given the crucial function of IR in the pathogenesis of MetS, it is beneficial to highlight the TyG index’s efficacy in screening MetS [25]. The TyG index is a valuable biomarker for IR due to its high sensitivity and specificity in comparison with the IR gold standard [16]. Given the TyG index’s advantage as a proxy for IR, it is biologically reasonable to investigate its relationship with breast cancer. Studies have already established the dysregulation of the TyG index in breast cancer [17–19]. Shi et al. [17]. included 11,466 participants and observed a positive interplay between the TyG index and high-risk breast cancer in the US adult population. Additionally, sufferers whose TyG index is in the highest quartile presented an intensified odds ratio versus the lowest after adjustment of various variates. Considering research conducted by Panigoro et al. [19]. in Indonesia, involving six public referral hospitals, it was revealed that BC sufferers had elevated values of the TyG index in comparison with healthy controls, which suggested a potential correlation between the TyG index and BC. Additionally, the study found a nonlinear dose-response interplay between the TyG index and BC, suggesting that the TyG index’s heightened value was linked to an intensified breast cancer risk. Alkurt et al. [18] engaged in a study displaying a substantial increase among breast cancer sufferers when compared to benign breast lesions. These findings were consistent with the cohort study, signifying that there’s an enhanced TyG index level among breast cancer sufferers than those with benign breast disease. Furthermore, an intensified TyG index was persistently relevant to a heightened breast cancer risk, regardless of the adjustment for various confounders.

There are several possible explanations for the mechanism underlying the carcinogenic role of the TyG index in the evolving process of breast cancer. Firstly, insulin resistance leads to hyperinsulinemia, which in turn motivates signaling pathways (Ras/MAPK and PI3K/Akt/mTOR), NF-κB nuclear translocation, and gene transcription from cancer-related genes. These molecular events ultimately contribute to breast cancer cell proliferation and survival by inhibiting apoptosis [26]. Additionally, excess insulin inhibits hepatic sex hormone-binding globulin synthesis, resulting in elevated estrogen bioactivity and promoting breast cancer progression [27]. Secondly, dyslipidemia and hyperglycemia are independent risk factors for developing BC and may exacerbate the occurrence of BC [28]. Breast cancer cells rely on increased glucose metabolism for energy production, and modified lipid metabolism also correlates with cancer cell signaling pathways [29]. To summarize, the enhanced TyG index observed in breast cancer sufferers may aggravate the evolving procedure of breast cancer depending on various mechanisms, including insulin signaling pathways, dyslipidemia, and altered energy metabolism.

As a result, it appears reasonable and feasible to combine triglycerides and blood glucose levels to measure their relationship with breast cancer. The TyG index, a proxy for IR, has shown a significant association with BC in this study. Interestingly, the TyG index was analyzed at different stages of BC, revealing significant variation in TyG index levels among stage 0, early stages, and advanced stages (*P* < 0.01). The heightened TyG index pertained to an enhanced risk of advanced BC, suggesting its potential as a non-invasive serum marker reflecting BC development and progression. Furthermore, the TyG index’s ability to distinguish between benign and each stage of breast cancer was assessed using ROC curves. The TyG index showed higher accuracy and specificity in predicting advanced stages with an AUC of 0.691 (sensitivity 75.56%, specificity 53.43%, *P* < 0.001), which provided strong evidence of the link between the TyG index and breast cancer occurrence and prognosis. It is recommended that the calculation and reporting of the TyG index be incorporated into routine biochemical testing to assess BC risk and alert oncologists to the potential prognosis of sufferers. Maintaining optimal levels of triglycerides and blood glucose, as well as successfully controlling the TyG index, is critical to minimizing breast cancer risk.

### Study strengths and limitations

This research has several strengths that make it significant. Firstly, it signified a potential correlation in the Chinese population of the TyG index and BC risk. Secondly, the TyG index was positively correlated with clinical stage, suggesting an interplay between the TyG index and breast cancer progression. Lastly, this study provided evidence that the TyG index had an independent relationship with an intensified breast cancer risk, even after adjusting for various confounders. These findings aligned with previous research and provided robust evidence for a potential link between insulin resistance and breast cancer incidence.

However, some limitations must also be acknowledged in the study. Firstly, sufficient information on important confounding factors such as dietary habits, fasting time, physical activity, age at first full-term pregnancy, breast density, and other potential factors could not be collected, which may have influenced the results. Secondly, information on whether all enrolled sufferers had recently taken glucose-lowering drugs or other lipid-lowering drugs, such as statins, which may influence TyG levels and potentially impact the outcomes of the study, was not gathered. Thirdly, the analysis is limited to a single center, posing threats to the widespread application of the results. Lastly, due to the retrospective nature of the research design, it was not possible to compare the TyG index with the IR gold standard method or the commonly used homeostasis model assessment, which are not typically included in routine preoperative breast cancer examinations.

## Conclusion

Overall, the study emphasizes the TyG index’s capability as an indicator for monitoring BC progression and as a predictive factor for BC risk. Integrating routine laboratory assessments for calculating the TyG index could help identify high-risk groups for breast cancer among the Chinese population with benign breast disease in clinical practice.

### Electronic supplementary material

Below is the link to the electronic supplementary material.

Supplementary Material 1

## Data Availability

The datasets generated and analyzed during the current study are available from the corresponding author on request.

## Abbreviations

BC: Breast cancer
TyG: Triglyceride-glucose
BBD: Benign breast disease
ROC: Receiver operating characteristic
MetS: Metabolic syndrome
IR: Insulin resistance
BG: Blood glucose
TC: Total cholesterol
TG: Triglyceride
HDL-c: High-density lipoprotein cholesterol
LDL-c: Low-density lipoprotein cholesterol
BMI: Body mass index
SD: Standard deviation
AUC: Area under the curve
OR: Odds ratio
CI: Confidence interval
CIS: Carcinoma in situ
Q: Quartile
