# Supplementary material for: Triglyceride-glucose index is a risk factor for breast cancer in China: a cross-sectional study
Source: Lipids Health Dis. 2024 Jan 26;23:29. doi: 10.1186/s12944-024-02008-0 (PMC10811843; doi:10.1186/s12944-024-02008-0)
Supplement: Supplementary file 1 — Supplementary Material 1 [file 12944_2024_2008_MOESM1_ESM.pdf]

Triglyceride-glucose index is a risk factor for breast cancer in China: a cross sectional study

by Cheng Jingwen

Submission date: 04-Jan-2024 04:29AM (UTC-0700)
Submission ID: 2266662354
File name: manuscript-revised20240104.docx (98.54K)
Word count: 3859
Character count: 22002

Triglyceride-glucose index is a risk factor for breast cancer in China: a cross-sectional study

Jinghua Zhang [1,#], Binbin Yin [1,#], Ya Xi [2], Yongying Bai [1,*]

Abstract

Background: This research delved into the association between the risk of the Chinese population suffering from breast cancer (BC) and the triglyceride-glucose (TyG) index.

Methods: A total of 2,111 sufferers with benign breast disease (BBD) and 477 sufferers with BC were enrolled, and their TyG index was measured. Participants with varying TyG index values were categorized into quartiles. Logistic regression analysis was employed to assess the relationship between the TyG index and BC risk. The diagnostic performance of the TyG index for different stages of BC was measured using the receiver operating characteristic (ROC) curve.

Results: The TyG index of BC sufferers exceeded that of BBD (P < 0.001). A continuous increase in the risk of BC was found to be positively correlated with an ever-increasing TyG index. In the unadjusted model, the risk of getting BC mounted with quartiles of the TyG index growing (P < 0.001). In a logistic regression analysis that included all confounders, the highest quartile of the TyG index was strongly linked to BC risk [1.43 (1.01, 2.02), P < 0.05]. Moreover, with the adjustment of potential confounders, a high TyG index was found to result in a 2.53-fold higher risk of being diagnosed with advanced BC.

Conclusions: The risen TyG index was positively correlated to the heightening risk of BC and had the potential to serve as a promising biomarker for BC. Individuals with a high TyG index ought to be mindful of the heightened risk of BC onset and progression.

Background

Breast cancer (BC) poses a significant public health challenge as it is the most common cancer globally [1]. In 2020 alone, it was estimated that the quantity of breast cancer cases would be around 2.3 million, with 685,000 deaths, and the previous projections suggested these numbers would continue to rise [2, 3]. Identifying modifiable risk factors and high-risk individuals is crucial to reducing the social impact of breast cancer. Despite significant advancements in breast cancer screening, diagnosis, therapy, and recurrence monitoring in the past few decades [4], the incidence rate of BC continues to increase [5]. Therefore, developing practical and reliable non-invasive markers that can identify the risk of symptomatic individuals is of significance to facilitate early diagnosis and reduce morbidity and mortality.

Metabolic syndrome (MetS), characterized by a range of biological factors such as obesity, dyslipidemia, dysglycemia, and hypertension, shows a close link to various types of diseases, including cancer [6, 7]. In the case of breast cancer, MetS has been linked to high-risk diseases and a poorer prognosis [8, 9]. Insulin resistance (IR), an important component of MetS, is indispensable in the evolving process of breast cancer [10]. However, the hyperinsulinemic-euglycemic clamp, which is the gold standard way to measure IR, is invasive, inefficient, labor-intensive, and technically challenging [11, 12], limiting its applicability in clinical settings.

Recently, the triglyceride-glucose (TyG) index has emerged as a promising object in medical research. This index, calculated from triglyceride level and glucose level, acts as a novel proxy for labeling IR [13]. It is practical, effective, and reproducible,

demonstrating good sensitivity and specificity in detecting IR [14-16]. Moreover, several researches have shown that the TyG index pertains to various cancers, and a positive correlation represented by the TyG index and BC risk has been notified in past studies conducted in Western populations and Southeast Asia [17-19]. It is worth noting that China accounts for 24% of newly diagnosed cases and 30% of BC deaths world-wide [20], and the median age of onset and diagnosis of BC is earlier than in Western countries and other Asian countries like Korea and Japan [21]. In conclusion, it is significant to consider unique genetic, environmental, and lifestyle factors in the Chinese population that may influence this relationship differently. Therefore, the research aimed to assess the plausible correlation between the TyG index and breast cancer related to the Chinese population, filling a significant research gap.

Methods

Study design and subjects

A retro analysis was made with 3,097 sufferers undergoing breast disease admitted to the Department of Surgery, Women's Hospital, Zhejiang University School of Medicine, from March 2020 to November 2021. The study received approval from the hospital's ethics committee (approval number: IRB-20210315-R), and informed consent was waived as anonymized sufferer records were used. However, certain individuals who meet the following criteria are excluded: (1) missing data on the TyG index, which consisted of triglyceride and blood glucose (BG) levels; (2) missing relevant demographic information or incomplete clinicopathological data; (3) duplicated data; (4) under 18 years of age; (5) male participants; (6) individuals who received surgical

treatments, chemotherapy, or radiotherapy before enrollment; (7) individuals with a history of cancer or autoimmune disorders; (8) individuals with conditions that may affect blood glucose levels, such as Cushing's syndrome, hyperthyroidism, polycystic ovary syndrome, diabetes mellitus, or pancreatitis; (9) individuals using fenofibrate triglyceride-lowering drugs; (10) pregnant or breastfeeding women.

The final analysis included a total of 2,588 individuals, comprising 2,111 cases of benign breast diseases (81.57%) and 477 cases of breast cancer (18.43%). The diagnosis of benign breast diseases or breast cancer in all enrolled sufferers was confirmed by postoperative pathology, and the staging guideline developed by the American Joint Committee on Cancer was exploited to determine the BC stages [22]. The screening details for the participants were illustrated in Fig. 1.

Data collection

The demographics of the participants were collected from the hospital information system, which included data on sex, age, weight, height, smoking, alcohol consumption, familial record of malignancy, age at menarche, hormonal contraception, and comorbidities. The laboratory information system provided measurements of total cholesterol (TC), triglyceride (TG), high-density lipoprotein cholesterol (HDL-c), low-density lipoprotein cholesterol (LDL-c), and blood glucose.

Laboratory analysis

The concentrations of TC, TG, HDL-c, LDL-c, and BG were analyzed utilizing an AU5800 chemistry analyzer (Beckman-Coulter, USA) in the hospital's clinical laboratory. The instrument underwent daily internal quality controls and annual

calibration to ensure accurate and reliable results. All procedures were executed in accordance with the instrument's standard operating procedures to maintain consistency and accuracy.

Definition of index

The formula for computing indexes was exemplified:

Body mass index (BMI) = weight (kg)/height squared (m^2). BMI was divided into underweight, normal weight, overweight, or obese, corresponding to < 18.5, 18.5-23.9, 24-28, and > 28 kg/m^2, respectively [23].

TyG index = Ln [TG (mg/dl) × BG (mg/dl)/2] [16].

Statistical analysis

Statistical analyses were acquired through IBM SPSS 20.0 (Chicago, USA), and figures were obtained through GraphPad Prism 8.0 (California, USA). Continuous variables in the study were illustrated using means ± standard deviations (means ± SD), while categorical variables were reported using frequencies and proportions (n, %). The independent-sample *t*-test, or one-way analysis of variance, was employed. The chi-square test was utilized for comparing categorical variables across groups. The diagnostic characteristics of the TyG index for different stages of BC were measured considering the receiver operating characteristic (ROC) curve and the accompanying area under the curve (AUC). Additionally, the nonlinearity of the dose-response curve was evaluated to explore the correlation between the TyG index and BC risk. Five knots were created at the 10th, 25th, 50th, 75th, and 90th percentiles, with the 50th percentile's TyG index as a reference point. Odds ratios (ORs) and 95% confidence

intervals (CIs) for each TyG index quartile were determined using analyses of logistic regression, with or without adjustments for potential covariates. $P < 0.05$ was denoted as statistical significance.

Results

Basic features of the enrolled sufferers

Table 1 presented the basic features of the enrolled sufferers. Among the 2,588 sufferers, the mean age was 42.17 ± 11.78 years, and the average BMI was 22.3 ± 3.0 kg/m^2. Out of the individuals affected, 477 sufferers were diagnosed with breast cancer, including 101 (21.17%) with carcinoma in situ (CIS) and 376 (78.83%) with invasive breast cancer. Significant intergroup discrepancies existed in the BC and BBD groups in terms of age, height, weight, BMI, TC, TG, LDL-c, BG, TyG index, and age at menarche (all $P < 0.001$). Additionally, the percentages of BMI category, TyG index category, smoking status, alcohol status, hypertension, and family history of cancer exhibited substantial disparities between the two groups (all $P < 0.05$). However, there was no intergroup difference in HDL-c levels or hormonal contraception rate (all $P > 0.05$).

The BC risk and the TyG index

Fig. 2 and Table 2 illustrated the link between the BC risk and the TyG index. Using the 50th percentile's TyG index as a reference, the results in Fig. 2 indicated a positive dose-response connection between BC risk and the TyG index in the total population. Crude and adjusted risks for the correlation of TyG index quartiles and BC risk were specified in Table 2. In the unadjusted Model 1, individuals' TyG index in the quartiles of 2nd (Q2), 3rd (Q3), and 4th (Q4) showed higher risks of BC compared to the first

quartile (Q1), with corresponding ORs (95% CI) of 1.81 (1.31, 2.51), 2.19 (1.59, 3.01), and 3.18 (2.34, 4.33), respectively.

In model 2, the Q4 of the TyG index showed a higher adjusted risk [1.50 (1.07, 2.11), $P < 0.05$] compared to the first quartile (Q1) after modifying age and BMI. Similarly, individuals in Q4 of the TyG index group in model 3 had a statistically significant higher risk, with an OR of [1.43 (1.01, 2.02), $P < 0.05$]. Model 3 included additional confounders regarding age, BMI, drinking, smoking, hypertension, family history of malignancy, age at menarche, and hormonal contraception.

The TyG index in the BC stages

Sufferers with advanced stages of the disease have a poorer prognosis. This study looked at the link between the TyG index and BC stages, which were categorized into stage 0 (carcinoma in situ), early stages (stages I + II), and advanced stages (stages III + IV). Fig. 3 demonstrated a climbing trend in the expression of the TyG index from carcinoma in situ to advanced stages of BC ($P < 0.01$). Additionally, risk analysis was conducted on various stages of BC, using stage 0 as the reference. Fig. 4 displayed the crude and adjusted ORs (95% CI) to investigate the correlation between the TyG index and the BC risk at various stages. In comparison with the TyG index for stage 0, the risk of advanced stages increased by 2.65 times in the unadjusted model (Fig. 4A). Similar results were obtained in models 2 (Fig. 4B) and 3 (Fig. 4C), which adjusted for age and BMI or included additional factors such as drinking status, smoking status, hypertension, family history of malignancy, age at menarche, and hormonal contraception.

Diagnostic characteristic of the TyG index for BC

Fig. 5 displayed the ROC curves for the TyG index in differentiating between benign and malignant breast diseases. The optimal threshold of the TyG index for BC was found to be 8.12 (AUC 0.608, sensitivity 71.91%, specificity 47.04%, $P < 0.001$) (Fig. 5A). The AUC for carcinoma in situ was 0.561 (sensitivity 35.64%, specificity 76.79%, $P < 0.05$) (Fig. 5B), while that for early stages was 0.611 (sensitivity 72.81%, specificity 47.75%, $P < 0.001$) (Fig. 5C). Interestingly, the TyG index of advanced stages had a greater maximum satisfactory accuracy and specificity than the other three groups, with an AUC of 0.691 (sensitivity 75.56%, specificity 53.43%, $P < 0.001$) (Fig. 5D).

Discussion

In this study, the aim was to delve into the correlation between the TyG index and breast cancer. This study included 2,111 sufferers with benign breast disease and 477 sufferers with breast cancer, making it the first comprehensive study concentrating on the connection between the TyG index and breast cancer in Chinese residents. BC sufferers had notable levels of the TyG index than those with BBD. Furthermore, a positive interplay between the TyG index and BC risk was found in the unadjusted model. Even after adjustments for various confounding factors, a significantly increased BC risk was observed in individuals in the uppermost quartile of the TyG index. Interestingly, the TyG index levels were positively correlated with the risk of advanced stages of breast cancer, suggesting its potential as a non-invasive serum marker reflecting breast cancer development and progression.

Epidemiological evidence increasingly indicates a correlation between MetS and the evolving process of breast cancer. Additionally, this association is linked to an

unfavorable treatment response, accelerated disease progression, and an unfavorable prognosis [8]. Insulin resistance, characterized by suppression of hepatic glucose manufacture and downward sensitivity to insulin-mediated glucose disposal [24], contributes greatly to the pathogenesis of MetS [10]. Given the crucial function of IR in the pathogenesis of MetS, it is beneficial to highlight the TyG index's efficacy in screening MetS [25]. The TyG index is a valuable biomarker for IR due to its high sensitivity and specificity in comparison with the IR gold standard [16]. Given the TyG index's advantage as a proxy for IR, it is biologically reasonable to investigate its relationship with breast cancer. Studies have already established the dysregulation of the TyG index in breast cancer [17-19]. Shi *et al*. [17] included 11,466 participants and observed a positive interplay between the TyG index and high-risk breast cancer in the US adult population. Additionally, sufferers whose TyG index is in the highest quartile presented an intensified odds ratio versus the lowest after adjustment of various variates. Considering research conducted by Panigoro *et al*. [19] in Indonesia, involving six public referral hospitals, it was revealed that BC sufferers had elevated values of the TyG index in comparison with healthy controls, which suggested a potential correlation between the TyG index and BC. Additionally, the study found a nonlinear dose-response interplay between the TyG index and BC, suggesting that the TyG index's heightened value was linked to an intensified breast cancer risk. Alkurt *et al*. [18] engaged in a study displaying a substantial increase among breast cancer sufferers when compared to benign breast lesions. These findings were consistent with the cohort study, signifying that there's an enhanced TyG index level among breast cancer sufferers than

those with benign breast disease. Furthermore, an intensified TyG index was persistently relevant to a heightened breast cancer risk, regardless of the adjustment for various confounders.

19

There are several possible explanations for the mechanism underlying the carcinogenic role of the TyG index in the evolving process of breast cancer. Firstly, insulin resistance leads to hyperinsulinemia, which in turn motivates signaling pathways (Ras/MAPK and PI3K/Akt/mTOR), NF-κB nuclear translocation, and gene transcription from cancer-related genes. These molecular events ultimately contribute to breast cancer cell proliferation and survival by inhibiting apoptosis [26]. Additionally, excess insulin inhibits hepatic sex hormone-binding globulin synthesis, resulting in elevated estrogen bioactivity and promoting breast cancer progression [27]. Secondly, dyslipidemia and hyperglycemia are independent risk factors for developing BC and may exacerbate the occurrence of BC [28]. Breast cancer cells rely on increased glucose metabolism for energy production, and modified lipid metabolism also correlates with cancer cell signaling pathways [29]. To summarize, the enhanced TyG index observed in breast cancer sufferers may aggravate the evolving procedure of breast cancer depending on various mechanisms, including insulin signaling pathways, dyslipidemia, and altered energy metabolism.

As a result, it appears reasonable and feasible to combine triglycerides and blood glucose levels to measure their relationship with breast cancer. The TyG index, a proxy for IR, has shown a significant association with BC in this study. Interestingly, the TyG index was analyzed at different stages of BC, revealing significant variation in TyG

index levels among stage 0, early stages, and advanced stages ($P < 0.01$). The heightened TyG index pertained to an enhanced risk of advanced BC, suggesting its potential as a non-invasive serum marker reflecting BC development and progression. Furthermore, the TyG index's ability to distinguish between benign and each stage of breast cancer was assessed using ROC curves. The TyG index showed higher accuracy and specificity in predicting advanced stages with an AUC of 0.691 (sensitivity 75.56%, specificity 53.43%, $P < 0.001$), which provided strong evidence of the link between the TyG index and breast cancer occurrence and prognosis. It is recommended that the calculation and reporting of the TyG index be incorporated into routine biochemical testing to assess BC risk and alert oncologists to the potential prognosis of sufferers. Maintaining optimal levels of triglycerides and blood glucose, as well as successfully controlling the TyG index, is critical to minimizing breast cancer risk.

Study strengths and limitations

This research has several strengths that make it significant. Firstly, it signified a potential correlation in the Chinese population of the TyG index and BC risk. Secondly, the TyG index was positively correlated with clinical stage, suggesting an interplay between the TyG index and breast cancer progression. Lastly, this study provided evidence that the TyG index had an independent relationship with an intensified breast cancer risk, even after adjusting for various confounders. These findings aligned with previous research and provided robust evidence for a potential link between insulin resistance and breast cancer incidence.

However, some limitations must also be acknowledged in the study. Firstly, sufficient

information on important confounding factors such as dietary habits, fasting time, physical activity, age at first full-term pregnancy, breast density, and other potential factors could not be collected, which may have influenced the results. Secondly, information on whether all enrolled sufferers had recently taken glucose-lowering drugs or other lipid-lowering drugs, such as statins, which may influence TyG levels and potentially impact the outcomes of the study, was not gathered. Thirdly, the analysis is limited to a single center, posing threats to the widespread application of the results. Lastly, due to the retrospective nature of the research design, it was not possible to compare the TyG index with the IR gold standard method or the commonly used homeostasis model assessment, which are not typically included in routine preoperative breast cancer examinations.

Conclusion

Overall, the study emphasizes the TyG index's capability as an indicator for monitoring BC progression and as a predictive factor for BC risk. Integrating routine laboratory assessments for calculating the TyG index could help identify high-risk groups for breast cancer among the Chinese population with benign breast disease in clinical practice.

References

1. Arnold M, Morgan E, Rumgay H, Mafra A, Singh D, Laversanne M, Vignat J, Gralow JR, Cardoso F, Siesling S, Soerjomataram I: Current and future burden of breast cancer: Global statistics for 2020 and 2040. Breast. 2022;66:15-23.
2. Sung H, Ferlay J, Siegel RL, Laversanne M, Soerjomataram I, Jemal A, Bray F: Global Cancer Statistics 2020: GLOBOCAN Estimates of Incidence and Mortality Worldwide for 36 Cancers in 185 Countries. CA Cancer J Clin. 2021;71:209-249.
3. Soerjomataram I, Bray F: Planning for tomorrow: global cancer incidence and the role of prevention 2020-2070. Nat Rev Clin Oncol. 2021;18:663-672.
4. Li X, Ma Z, Mei L: Cuproptosis-related gene SLC31A1 is a potential predictor for diagnosis, prognosis and therapeutic response of breast cancer. Am J Cancer Res. 2022;12:3561-3580.
5. Britt KL, Cuzick J, Phillips KA: Key steps for effective breast cancer prevention. Nat Rev Cancer. 2020;20:417-436.
6. Bishehsari F, Voigt RM, Keshavarzian A: Circadian rhythms and the gut microbiota: from the metabolic syndrome to cancer. Nat Rev Endocrinol. 2020;16:731-739.
7. Bellastella G, Scappaticcio L, Esposito K, Giugliano D, Maiorino MI: Metabolic syndrome and cancer: "The common soil hypothesis". Diabetes Res Clin Pract. 2018;143:389-397.
8. Dong S, Wang Z, Shen K, Chen X: Metabolic Syndrome and Breast Cancer: Prevalence, Treatment Response, and Prognosis. Front Oncol. 2021;11:629666.
9. Buono G, Crispo A, Giuliano M, De Angelis C, Schettini F, Forestieri V, Lauria R, De Laurentiis M, De Placido P, Rea CG, et al: Metabolic syndrome and early stage breast cancer outcome: results from a prospective observational study. Breast Cancer Res Treat. 2020;182:401-409.
10. Biello F, Platini F, D'Avanzo F, Cattrini C, Mennitto A, Genestroni S, Martini V, Marzullo P, Aimaretti G, Gennari A: Insulin/IGF Axis in Breast Cancer: Clinical Evidence and Translational Insights. Biomolecules. 2021;11.
11. Singh B, Saxena A: Surrogate markers of insulin resistance: A review. World J Diabetes. 2010;1:36-47.
12. DeFronzo RA, Tobin JD, Andres R: Glucose clamp technique: a method for quantifying insulin secretion and resistance. Am J Physiol. 1979;237:E214-223.
13. Simental-Mendia LE, Rodriguez-Moran M, Guerrero-Romero F: The product of fasting glucose and triglycerides as surrogate for identifying insulin resistance in apparently healthy subjects. Metab Syndr Relat Disord. 2008;6:299-304.
14. Tian X, Zuo YT, Chen SH, Liu Q, Tao BN, Wu SL, Wang AX: Triglyceride-glucose index is associated with the risk of myocardial infarction: an 11-year prospective study in the Kailuan cohort. Cardiovascular Diabetology. 2021;20.
15. Navarro-Gonzalez D, Sanchez-Inigo L, Pastrana-Delgado J, Fernandez-Montero A, Martinez JA: Triglyceride-glucose index (TyG index) in

comparison with fasting plasma glucose improved diabetes prediction in patients with normal fasting glucose: The Vascular-Metabolic CUN cohort. Prev Med. 2016;86:99-105.

16. Guerrero-Romero F, Simental-Mendia LE, Gonzalez-Ortiz M, Martinez-Abundis E, Ramos-Zavala MG, Hernandez-Gonzalez SO, Jacques-Camarena O, Rodriguez-Moran M: The product of triglycerides and glucose, a simple measure of insulin sensitivity. Comparison with the euglycemic-hyperinsulinemic clamp. J Clin Endocrinol Metab. 2010;95:3347-3351.
17. Shi H, Zhou L, Yang S, Zhou H: The relationship between Triglyceride and glycose (TyG) index and the risk of gynaecologic and breast cancers. Clin Nutr ESPEN. 2022;51:345-352.
18. Alkurt EG, Ozkan MB, Turhan VB: Predictive value of triglyceride/glucose index (TyG) in predicting breast cancer in patients with breast mass. Eur Rev Med Pharmacol Sci. 2022;26:4671-4676.
19. Panigoro SS, Sutandyo N, Witjaksono F, Siregar NC, Ramli R, Hariani R, Pangarsa EA, Prajoko YW, Puruhita N, Hamdani W, et al: The Association Between Triglyceride-Glucose Index as a Marker of Insulin Resistance and the Risk of Breast Cancer. Front Endocrinol (Lausanne). 2021;12:745236.
20. Cao W, Chen HD, Yu YW, Li N, Chen WQ: Changing profiles of cancer burden worldwide and in China: a secondary analysis of the global cancer statistics 2020. Chin Med J (Engl). 2021;134:783-791.
21. Tao X, Li T, Gandomkar Z, Brennan PC, Reed WM: Incidence, mortality, survival, and disease burden of breast cancer in China compared to other developed countries. Asia Pac J Clin Oncol. 2023;19:645-654.
22. Giuliano AE, Connolly JL, Edge SB, Mittendorf EA, Rugo HS, Solin LJ, Weaver DL, Winchester DJ, Hortobagyi GN: Breast Cancer-Major changes in the American Joint Committee on Cancer eighth edition cancer staging manual. CA Cancer J Clin. 2017;67:290-303.
23. Chen C, Lu FC, Department of Disease Control Ministry of Health PRC: The guidelines for prevention and control of overweight and obesity in Chinese adults. Biomed Environ Sci. 2004;17 Suppl:1-36.
24. Gutch M, Kumar S, Razi SM, Gupta KK, Gupta A: Assessment of insulin sensitivity/resistance. Indian J Endocrinol Metab. 2015;19:160-164.
25. Nabipoorashra SA, Seyedi SA, Rabizadeh S, Ebrahimi M, Ranjbar SA, Reyhan SK, Meysamie A, Nakhjavani M, Esteghamati A: The accuracy of triglyceride-glucose (TyG) index for the screening of metabolic syndrome in adults: A systematic review and meta-analysis. Nutrition Metabolism and Cardiovascular Diseases. 2022;32:2677-2688.
26. Rose DP, Vona-Davis L: The cellular and molecular mechanisms by which insulin influences breast cancer risk and progression. Endocrine-Related Cancer. 2012;19:R225-R241.
27. Kim DS, Scherer PE: Obesity, Diabetes, and Increased Cancer Progression. Diabetes Metab J. 2021;45:799-812.
28. Kang CF, LeRoith D, Gallagher EJ: Diabetes, Obesity, and Breast Cancer.

Endocrinology. 2018;159:3801-3812.
29. Stone TW, McPherson M, Gail Darlington L: Obesity and Cancer: Existing and New Hypotheses for a Causal Connection. EBioMedicine. 2018;30:14-28.

Triglyceride-glucose index is a risk factor for breast cancer in China: a cross sectional study

ORIGINALITY REPORT

16%	12%	15%	1%
SIMILARITY INDEX	INTERNET SOURCES	PUBLICATIONS	STUDENT PAPERS

PRIMARY SOURCES

1	cardiab.biomedcentral.com<br>Internet Source	3%
2	www.frontiersin.org<br>Internet Source	1%
3	Haimeng Shi, Feifei Guo, Kang Zheng, Rong Li, Huaijun Zhou. " Triglyceride-glucose index (index) and endometrial carcinoma risk: A retrospective cohort study ", International Journal of Gynecology & Obstetrics, 2023<br>Publication	1%
4	www.grafiati.com<br>Internet Source	1%
5	Luli Xu, Mingyang Wu, Shuohua Chen, Yingping Yang, Youjie Wang, Shouling Wu, Yaohua Tian. "Triglyceride–glucose index associates with incident heart failure: a cohort study", Diabetes & Metabolism, 2022<br>Publication	1%
6	bmcwomenshealth.biomedcentral.com<br>Internet Source	1%

7	www.alliedacademies.org<br>Internet Source	1%
8	Ru Zhang, Qing Guan, Mengting Zhang, Yajie Ding et al. "Association Between Triglyceride-Glucose Index and Risk of Metabolic Dysfunction-Associated Fatty Liver Disease: A Cohort Study", Diabetes, Metabolic Syndrome and Obesity: Targets and Therapy, 2022<br>Publication	1%
9	Su Zou, Yingjia Xu. "Association of the triglyceride-glucose index and major adverse cardiac and cerebrovascular events in female patients undergoing percutaneous coronary intervention with drug-eluting stents: A retrospective study", Diabetes Research and Clinical Practice, 2021<br>Publication	1%
10	Yue Wang, Yue Wang, Shuaifeng Sun, Xinyan Liu, Wenxin Zhao, Wenzheng Li, Min Suo, Zheng Wu, Xiaofan Wu. "Triglyceride-glucose index level and variability and outcomes in patients with acute coronary syndrome undergoing percutaneous coronary intervention: an observational cohort study", Lipids in Health and Disease, 2022<br>Publication	1%

11	Shutong Dong, Zehao Zhao, Xin Huang, Meishi Ma, Zhiqiang Yang, Chu Fan, Hongya Han, Zhijian Wang, Dongmei Shi, Yujie Zhou. "Triglyceride-glucose index is associated with poor prognosis in acute coronary syndrome patients with prior coronary artery bypass grafting undergoing percutaneous coronary intervention", Cardiovascular Diabetology, 2023<br>Publication	<1 %
12	econtent.hogrefe.com<br>Internet Source	<1 %
13	www.researchgate.net<br>Internet Source	<1 %
14	Jin-Wen Luo, Wen-Hui Duan, Yan-Qiao Yu, Lei Song, Da-Zhuo Shi. "Prognostic Significance of Triglyceride-Glucose Index for Adverse Cardiovascular Events in Patients With Coronary Artery Disease: A Systematic Review and Meta-Analysis", Frontiers in Cardiovascular Medicine, 2021<br>Publication	<1 %
15	Shi Tai, Liyao Fu, Ningjie Zhang, Rukai Yang, Yuying Zhou, Zhenhua Xing, Yongjun Wang, Shenghua Zhou. "Association of the cumulative triglyceride-glucose index with major adverse cardiovascular events in	<1 %

patients with type 2 diabetes", Cardiovascular Diabetology, 2022
Publication

16	Jiaye Zhang, Linlin Jia, Tongying Zhu, Hao Zhu, Li Shu. "The relationship and interaction between triglyceride glucose index and obesity in the risk of prehypertension population: a cross-sectional study from a survey in Anhui, Eastern China", BMC Cardiovascular Disorders, 2023<br>Publication	<1 %
17	www.dovepress.com<br>Internet Source	<1 %
18	Anran Wang, Yapeng Li, Lue Zhou, Kai Liu et al. "Triglyceride-Glucose Index Is Related to Carotid Plaque and Its Stability in Nondiabetic Adults: A Cross-Sectional Study", Frontiers in Neurology, 2022<br>Publication	<1 %
19	Yue Zhang, Xiaosong Ding, Bing Hua, Qingbo Liu, Hui Gao, Hui Chen, Xue-Qiao Zhao, Weiping Li, Hongwei Li. "High Triglyceride-Glucose Index is Associated with Poor Cardiovascular Outcomes in Nondiabetic Patients with ACS with LDL-C below 1.8 mmol/L", Journal of Atherosclerosis and Thrombosis, 2021<br>Publication	<1 %

20	journals.lww.com<br>Internet Source	<1%
21	Mingxiao Guo, Mengdi Li, Fengtao Cui, Xinping Ding, Wei Gao, Xingqiang Fang, Li Chen, Hanyun Wang, Piye Niu, junxiang ma. "MTBE exposure may increase the risk of insulin resistance in male gas station workers", Environmental Science: Processes & Impacts, 2024<br>Publication	<1%
22	Wei-Yu Su, Szu-Chia Chen, Yu-Ting Huang, Jiun-Chi Huang, Pei-Yu Wu, Wei-Hao Hsu, Mei-Yueh Lee. "Comparison of the Effects of Fasting Glucose, Hemoglobin A1c, and Triglyceride–Glucose Index on Cardiovascular Events in Type 2 Diabetes Mellitus", Nutrients, 2019<br>Publication	<1%
23	assets.researchsquare.com<br>Internet Source	<1%
24	bmcpublichealth.biomedcentral.com<br>Internet Source	<1%
25	dmsjournal.biomedcentral.com<br>Internet Source	<1%
26	pubmed.ncbi.nlm.nih.gov<br>Internet Source	<1%

27 R., Vinitha K.. "A Comparative Study of Serum Insulin and Insulin Resistance in Patients with Breast Carcinoma Andhealthy Subjects", Rajiv Gandhi University of Health Sciences (India), 2023
Publication
<1%

28 Seyed Ali Nabipoorashrafi, Seyed Arsalan Seyedi, Soghra Rabizadeh, Menooa Ebrahimi et al. "The Accuracy of Triglyceride-Glucose (TyG) index for the Screening of Metabolic Syndrome in adults: A Systematic Review and Meta-Analysis", Nutrition, Metabolism and Cardiovascular Diseases, 2022
Publication
<1%

29 Betsabe Contreras-Haro, Sandra Ofelia Hernandez-Gonzalez, Laura Gonzalez-Lopez, Maria Claudia Espinel-Bermudez et al. "Fasting triglycerides and glucose index: a useful screening test for assessing insulin resistance in patients diagnosed with rheumatoid arthritis and systemic lupus erythematosus", Diabetology & Metabolic Syndrome, 2019
Publication
<1%

30 Haipeng Yao, Zhen Sun, Wei Yuan, Chen Shao, Honghua Cai, Lihua Li, Yongjiang Qian, Zhongqun Wang. "Relationship Between the Triglyceride-Glucose Index and Type 2
<1%

Diabetic Macroangiopathy: A Single-Center Retrospective Analysis", Diabetes, Metabolic Syndrome and Obesity: Targets and Therapy, 2022
Publication

31	Huancong Zheng, Guanzhi Chen, Kuangyi Wu, Weiqiang Wu et al. "Relationship between cumulative exposure to triglyceride-glucose index and heart failure: a prospective cohort study", Cardiovascular Diabetology, 2023<br>Publication	<1 %
32	Panya Chamroonkiadtikun, Thareerat Ananchaisarp, Worawit Wanichanon. "The triglyceride-glucose index, a predictor of type 2 diabetes development: A retrospective cohort study", Primary Care Diabetes, 2020<br>Publication	<1 %

Exclude quotes	Off	Exclude matches	Off
Exclude bibliography	On		
